# A New Light on the Evolution and Propagation of Prehistoric Grain Pests: The World's Oldest Maize Weevils Found in Jomon Potteries, Japan

**DOI:** 10.1371/journal.pone.0014785

**Published:** 2011-03-29

**Authors:** Hiroki Obata, Aya Manabe, Naoko Nakamura, Tomokazu Onishi, Yasuko Senba

**Affiliations:** 1 Faculty of Letters, Kumamoto University, Kumamoto City, Kumamoto Prefecture, Japan; 2 Faculty of Law and Letters, Kagoshima University, Kagoshima City, Kagoshima Prefecture, Japan; 3 Faculty of Intercultural Studies, The International University of Kagoshima, Kagoshima City, Kagoshima Prefecture, Japan; 4 Project for Seed Impression Studies, Kumamoto University, Kumamoto City, Kumamoto Prefecture, Japan; American Museum of Natural History, United States of America

## Abstract

Three *Sitophilus* species (*S. granarius* L., *S. oryzae* L., and *S. zeamais* Mots.) are closely related based on DNA analysis of their endosymbionts. All are seed parasites of cereal crops and important economic pest species in stored grain. The *Sitophilus* species that currently exist, including these three species, are generally believed to be endemic to Asia's forested areas, suggesting that the first infestations of stored grain must have taken place near the forested mountains of southwestern Asia. Previous archaeological data and historical records suggest that the three species may have been diffused by the spread of Neolithic agriculture, but this hypothesis has only been established for granary weevils in European and southwestern Asian archaeological records. There was little archeological evidence for grain pests in East Asia before the discovery of maize weevil impressions in Jomon pottery in 2004 using the “impression replica” method. Our research on Jomon agriculture based on seed and insect impressions in pottery continued to seek additional evidence. In 2010, we discovered older weevil impressions in Jomon pottery dating to ca. 10 500 BP. These specimens are the oldest harmful insects in the world discovered at archaeological sites. Our results provide evidence of harmful insects living in the villages from the Earliest Jomon, when no cereals were cultivated. This suggests we must reconsider previous scenarios for the evolution and propagation of grain pest weevils, especially in eastern Asia. Although details of their biology or the foods they infested remain unclear, we hope future interdisciplinary collaborations among geneticists, entomologists, and archaeologists will provide the missing details.

## Introduction

Granary (*Sitophilus granarius* L.), rice (*Sitophilus oryzae* L.), and maize (*Sitophilus zeamais* Mots.) weevils, known as “snout weevils”, feed inside rice or barley grains during their larval stage and pupate inside the grains [1]. Rice and maize weevils are widespread in warm regions. In Europe and North America, they are replaced by temperate species such as the granary weevil [2]. The global distribution of these insects occurred relatively recently, and they have continued to spread as a result of worldwide cereal trading during the 20th century.

In archaeological records from Europe, the Mediterranean, and Asia, the oldest granary weevil discovery has been dated to ca. 7000 BP. The granary weevils recovered from many sites in these regions correspond to the diffusion of Neolithic agriculture or Roman cereal trading and transport of soldiers. In contrast, few prehistoric discoveries of the rice weevil and maize weevil had been reported in China and Japan by 2003. In general, the granary weevil and its two sister species are monophyletic species in genus *Sitophilus* and are believed to have originated in Asia [3].

However, many impressions of cereal weevils have been discovered in Jomon pottery in Japan since researchers first applied the “impression replica” method in 2004. This method involves making a model of the original form that created the cavity in a potsherd. Since 2004, the number of impression records has gradually increased. Until 2009, we had collected 37 samples, of which the oldest dated to ca. 4500 BP. However, our recent search for seed or insect impressions at archeological sites on Tanegashima Island in spring 2010 provided many new records, which suggest that the association between maize weevils and humans occurred in the Earliest Jomon (11 500 to 7300 BP), at ca. 10 500 BP. This discovery suggests that researchers must reconsider previous scenarios for the evolution and propagation of grain pest weevils, their adaptation to grain storage systems, and their spread along with prehistoric agriculture, especially in eastern Asia. Our new results suggest that eastern Asian grain pests, including the maize weevil and probably the rice weevil, evolved differently from the granary weevil in Europe.

The goal of this paper is to introduce our new discovery and discuss its preliminary significance for archaeological research in eastern Asia.

## Results and Discussion

### Discovery of weevil impressions in Jomon potsherds

Greek and Roman records from ca. 2200 BP describe weevils infesting wheat. In China, the oldest record appears in the “Ěryă” dictionary, which was published between ca. 2500 to 2200 BP. By this time, weevils were already recognized as storage pests that infested rice. The first Japanese description of maize weevils appears in historical records from ca. 1000 BP [4], [5].

Archaeological records from Europe and southwestern Asia reveal that granary weevils had spread along with the diffusion of agriculture and grain trading or transport between ca. 7000 BP and ca. 1900 BP [3]. For example, granary weevils were discovered in a funeral offering of barley to a dead king of the Egyptian Sixth Dynasty (ca. 4300 BP). In China, there is little archaeological evidence of prehistoric grain pests. The only example is of rice weevils infesting barley in a grave from the Han Dynasty (2118 BP) [4], [5].

In Japanese archeological records, maize weevil impressions were first discovered in Jomon pottery in 2004. Before then, the oldest specimens (beetle carapaces) came from a ditch deposit at the Ikegamisone site (ca. 2000 BP). The beetle carapaces of maize weevils were also recovered at the Fujiwara Palace site (ca.1190 BP) and the Kiyosu Castle site (ca. 390 BP) [6]. In 2004, maize weevil impressions were discovered (ca. 3500 BP) in the Late Jomon (4500 to 3300 BP) pottery on Kyushu [7]. Another recent discovery revealed soybean and adzuki bean cultivation during the Early Jomon to the Latest Jomon [8], [9]. These beans were also discovered in Jomon pottery as impressions.

**Figure 1:**
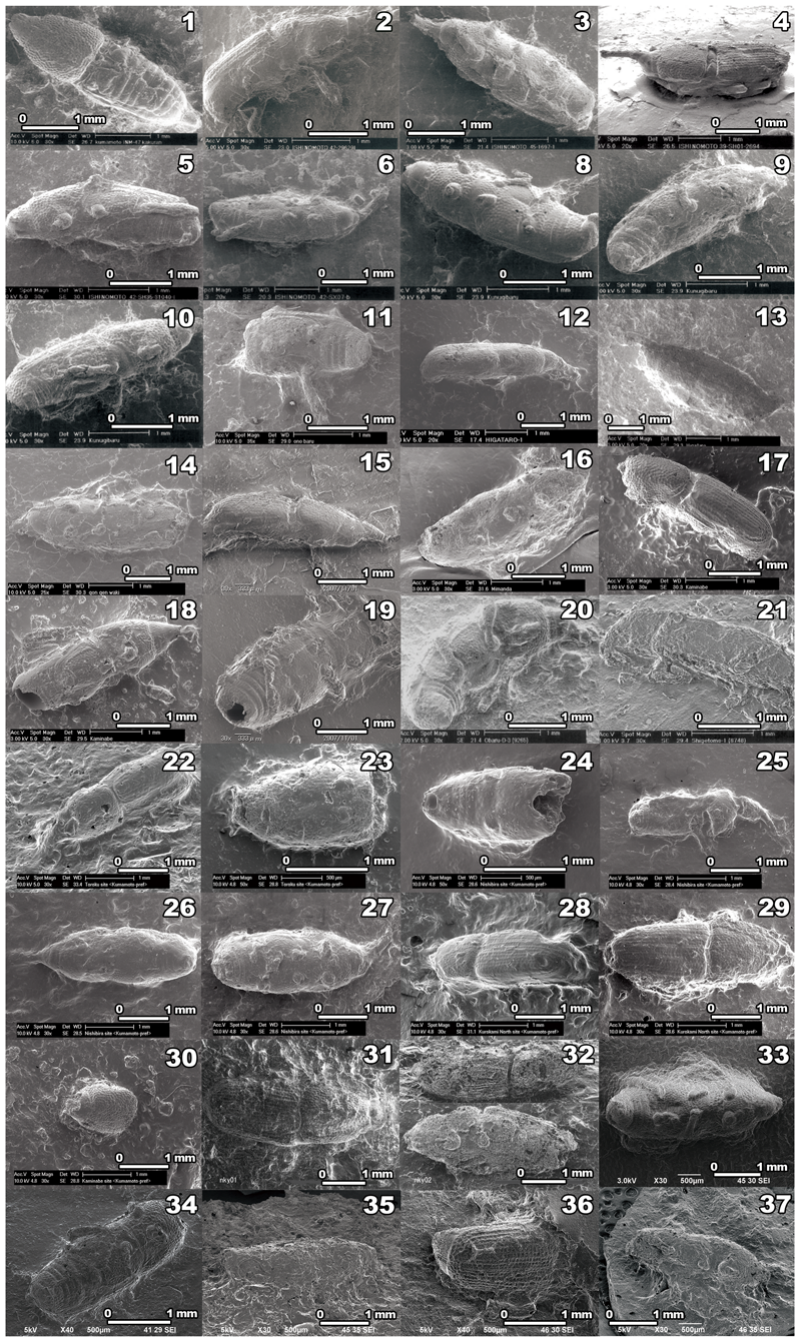
Scanning electron micrograph (SEM) images of impression replicas of maize weevils that had been discovered at Jomon sites by 2009. The 37 weevil impressions were discovered in Jomon pottery, mainly collected on Kyushu, dating to between ca. 4500 and ca. 3000 BP. Based on the diagnostic criteria described in the text, including the size of the specimens, these weevils appear to have been maize weevils (*Sitophilus zeamais*). Details of each impression replica are provided in Table 1.

Subsequently, many maize weevil impressions have been discovered at the Late Jomon sites, especially on Kyushu. In 2007, two maize weevil impressions were discovered at the Latest Jomon (3300 to 3000 BP) site in Yamanashi Prefecture [10]. By 2008, 32 weevil impressions had been found at 13 sites (Fig. 1, Table 1; samples 1 to 32). Most impressions appeared to be of maize weevils, based on their size and anatomical characteristics. These new discoveries suggest that maize weevils might have coexisted with humans as early as the Late Jomon period. The earliest impression was found inside a Kanezaki 3–type deep bowl dating to ca. 4000 BP. The number of specimens increases rapidly after the Nishibira pottery phase (ca. 3800 BP), with a peak during the last quarter of the Late Jomon [11]. Subsequently, impression discovery rates remain stable. We discovered three new impressions at early the Late Jomon sites in Kagoshima Prefecture in 2009. This finding dates the appearance of weevils 200 or 300 years earlier than previous research (Fig. 1, Table 1; samples 35-37).

**Table 1:** Details of the maize weevil impressions that were obtained at Jomon sites by 2009. Numbers correspond to the photographs in Figure 1.

	Part of insect covered by the impression	Site	Sample name	Type of pottery	Shape/part	Phase during the Jomon	Estimated age (BP)
1	Dorsal view (missing rostrum, wings, and legs)	Ishinomoto(Kumamoto Pref.)	INM-47	Amagi	Deep bowl/rim	The end of the Late J.	ca. 3450
2	Side view (missing legs)	Ishinomoto(Kumamoto Pref.)	42-29629-1	Amagi	Deep bowl/rim	The end of the Late J.	ca. 3450
3	Ventral view (missing rostrum and legs)	Ishinomoto(Kumamoto Pref.)	45-1697-1	Goryo	Shallow bowl/rim	The latter half of the Late J.	ca. 3500
4	Dorsal view (missing legs)	Ishinomoto(Kumamoto Pref.)	39-SH01-2694	Koga	Shallow bowl/body	The end of the Late J.	ca. 3400
5	Ventral view (missing rostrum and legs)	Ishinomoto(Kumamoto Pref.)	47-SH35-31040-1	Amagi	Deep bowl/unknown	The end of the Late J.	ca. 3450
6	Ventral view (missing legs)	Ishinomoto(Kumamoto Pref.)	47-SX-07-b	Goryo	Shallow bowl/unknown	Last half of the Late	ca. 3500
7	Unknown	Ishinomoto (Kumamoto Pref.)	Unknown	Unknown	Unknown	The Latest J.	ca. 3400 to 3000
8	Ventral view (missing rostrum and legs)	Kunugibaru(Kagoshima Pref.)	Kunugibaru-1	Unknown	Deep bowl/rim	The first half of the Latest J.	ca. 3200 to 3000
9	Side view (missing rostrum and legs)	Kunugibaru(Kagoshima Pref.)	Kunugibaru-2	Unknown	Deep bowl/rim	The first half of the Latest J.	ca. 3200 to 3000
10	Side view (missing rostrum and legs)	Kunugibaru(Kagoshima Pref.)	Kunugibaru-3	Unknown	Deep bowl/rim	The first half of the Latest J.	ca. 3200 to 3000
11	Ventral view (missing thorax and legs)	Ohnobaru(Nagasaki Pref.)	ONB1010	Tarozako	Deep bowl/base	The end of the Late J.	ca. 3600
12	Side view (missing legs)	Higataro(Nagasaki Pref.)	HIG115	Kurokawa?	Deep bowl/body	The first half of the Latest J.	ca. 3300
13	Side view (missing legs)	Higataro(Nagasaki Pref.)	10381-03	Kurokawa?	Deep bowl/body	The first half of the Latest J.	ca. 3300
14	Ventral view (missing rostrum and legs)	Gongenwaki(Nagasaki Pref.)	GGW-021	New Kurokasa	Bowl/body	The middle of the Latest J.	ca. 3000
15	Side view (missing rostrum and legs)	Ohnobaru(Nagasaki Pref.)	ONB1018	Tarozako?	Deep bowl/body	The latter half of the Late J.	ca. 3600
16	Ventral view (missing rostrum and legs)	Mimanda(Kumamoto Pref.)	MD0019	Tarozako	Bowl/base	The middle of the Late J.	ca. 3600
17	Side view (missing head and legs)	Kaminabe(Kumamoto Pref.)	KNB05	Amagi	Deep bowl/rim	The end of the Late J.	ca. 3450
18	Side view (missing head and legs)	Kaminabe(Kumamoto Pref.)	KNB32	Koga?	Deep bowl/body	The end of the Late J.	ca. 3400
19	Side view (missing legs)	Kaminabe(Kumamoto Pref.)	KNB34	Amagi?	Deep bowl/body	The end of the Late J.	ca. 3450
20	Side view (missing rostrum and legs)	Ohbaru D(Fukuoka Pref.)	Ohbaru-D-3 (9265)	Unknown	Deep bowl/unknown	The first half of the Latest J.	ca. 3200 to 3000
21	Side view (missing rostrum and legs)	Shigetome(Fukuoka Pref.)	Shigetome1 (8748)	Amagi	Deep bowl/body	The end of the Late J.	ca. 3450
22	Dorsal view (missing legs)	Toroku shell midden.(Kumamoto Pref.)	TR11	Kanezaki 3	Deep bowl/body	The first half of the Late J.	ca. 4000
23	Dorsal view (missing rostrum and trunk)	Toroku shell midden(Kumamoto Pref.)	TR21	Mimanda	Deep bowl/body	The latter half of the Late J.	ca. 3550
24	Ventral view (a trunk)	Nishibira shell midden(Kumamoto Pref.)	NB02	Nishibira	Bowl/body	The first half of the Late J.	ca. 3700
25	Side view (missing legs)	Nishibira shell midden(Kumamoto Pref.)	NB07	Nishibira?	Bowl/body	The first half of the Late J.	ca. 3700
26	Dorsal view (missing legs)	Nishibira shell midden.(Kumamoto Pref.)	NB08	Nishibira?	Bowl/body	The first half of the Late J.	ca. 3700
27	Ventral view (missing legs)	Nishibira shell midden(Kumamoto Pref.)	NB17	Goryou	Shallow bowl/rim	The latter half of the Late J.	ca. 3500
28	Dorsal view (missing rostrum and legs)	Kurokamimachi(Kumamoto Pref.)	KKN07	Unknown	Deep bowl/body	The latter half of the Late J.?	ca. 3600 to 3400
29	Dorsal view (missing rostrum and legs)	Kurokamimachi(Kumamoto Pref.)	KKN08	Unknown	Deep bowl/body	The latter half of the Late J.?	ca. 3600 to 3400
30	Dorsal view (a thorax)	Kaminabe(Kumamoto Pref.)	KNB22	Amagi?	Deep bowl/body	The end of the Late J.	ca. 3450
31	Dorsal view (missing rostrum and legs)	Nakaya(Yamanashi Pref.)	Nky01	Shimizutennouzan	Deep bowl/body	The first half of the Latest J.	ca. 3000
32	Side view (missing rostrum and legs)	Nakaya(Yamanashi Pref.)	Nky02	Shimizutennouzan	Deep bowl/body	The first half of the Latest J.	ca. 3000
33	Ventral view (missing rostrum and legs)	Mimanda(Kumamoto Pref.)	MMD2054	Unknown	Deep bowl/body	The latter half of the Late J.?	ca. 3600 to 3400
34	Ventral view (missing head and legs)	Ohnobaru(Nagasaki Pref.)	ONB1116	Unknown	Deep bowl/body	The latter half of the Late J.?	ca. 3600 to 3400
35	Ventral view (missing rostrum and legs)	Kakiuchi(Kagoshima Pref.)	KKU0019	Namiki-Nanpukuji	Deep bowl/body	The beginning of the Late J.	ca. 4500 to 4000
36	Dorsal view (abdomen)	Izumi shell midden(Kagoshima Pref.)	KZK0008	Izumi	Deep bowl/rim	The beginning of the Late J.	ca. 4300
37	Side view (missing rostrum and legs)	Nanbarauchibori(Kagoshima Pref.)	NBU0005	Ichiki	Deep bowl/rim	The beginning of the Late J.	ca. 4200

Japanese academic circles on archaeology divides the Jomon Period into six phases as followings. The Incipient Jomon (15000-11500BP), The Earliest Jomon (11500-7300BP), The Early Jomon (7300-5500BP), The Middle Jomon (5500-4500BP), The Late Jomon (4500-3300BP), The Latest Jomon (3300-3000BP).

### Old hypothesis: Maize weevils document the origins of rice cultivation in Japan

Maize weevils feed on stored grain and on fruits such as peach or apple in North America, and also inhabit forests or grasslands in southern Japan, where they feed on flowers in the spring. The adult weevils feed on 37 families and 96 species of plants, but the larvae feed on only 11 families and 31 species [2]. The adult maize weevils from Kumamoto City that we tested preferred large grains such as rice, barley, and wheat. They did not feed on adzuki bean, hemp, or rice in the husk. The weevils also infested acorns, but only those without seed coats, where they successfully matured [11]. However, during the Jomon period, acorns were generally stored with their seed coats to protect them from decay, so few or no maize weevils would have infested acorns during this period [12]. Although adult maize weevils feed on rice flour, they never oviposit there [13].

Two hypotheses have been proposed to explain the diffusion of rice cultivation into Japan. First, this form of agriculture may have diffused to Japan from the Shantong Peninsula through Korea ca. 4000 BP [11], [14]; alternatively, it may have diffused from southern China through the Ryukyu Islands ca. 6700 BP [15]. The former hypothesis is accepted by most archaeologists. Evidence from archaeological sites dating to those times (charred seeds, seed impressions, and phytoliths) suggests the cultivation of cereals in the Poaceae (e.g., rice, barley), which were introduced to Kyushu from Korea. The second hypothesis is not supported by archeological or archaeobotanical evidence [16], [17]. The earliest rice cultivation on the Ryukyu Islands occurred in ca. 1100 BP and was not introduced from southern China; instead, it was introduced at the time of human immigration from Kyushu into the region [11], [14].

If the maize weevil depends on crop cultivation, then it should have appeared at roughly the same time as the expansion of agriculture, which is known from Japanese archaeobotanical records, and indeed, archaeological artifacts suggest increasingly strong cultural relationships between southern Korea and northwestern Kyushu at this time. Furthermore, rice phytoliths have been recovered from the Late Jomon pottery in northwestern Kyushu. The presence of maize weevils therefore suggests the existence of rice or barley cultivation during the Late to the Latest Jomon periods, and that they invaded Japan from Korea, accompanying rice cultivation [11].

### New evidence: older maize weevil impressions

We have new evidence that contradicts the original hypothesis. In February 2010, we discovered the oldest impressions of maize weevils, which we obtained from the Earliest Jomon potsherds dated to ca. 10 500 BP from the Sanbonmatsu (SBM) site in Kagoshima Prefecture. The site, which is on a terrace (50 m asl) on the eastern coast of Tanagashima Island, 40 km southeast of the Ohsumi Peninsula, was excavated in 2007 in a project organized by the Nishinoomote City Board of Education. Researchers discovered cultural layers containing many artifacts, mainly from the Earliest Jomon period.

When we examined the potsherds to find seed and other impressions in February 2010, we found two fragments that contained maize weevil impressions. During our second examination, in April 2010, we found five fragments with maize weevil impressions. These fragments came from Yoshida-type deep bowls and were ornamented with shells, a popular ornamentation during the first half of the Earliest Jomon period in southern Kyushu [18]. This cluster has ^14^C dates from ca. 9240 BP to ca. 9330 BP, suggesting that the Yoshida pottery dates to ca. 10 500 cal BP [19].

**Figure 2:**
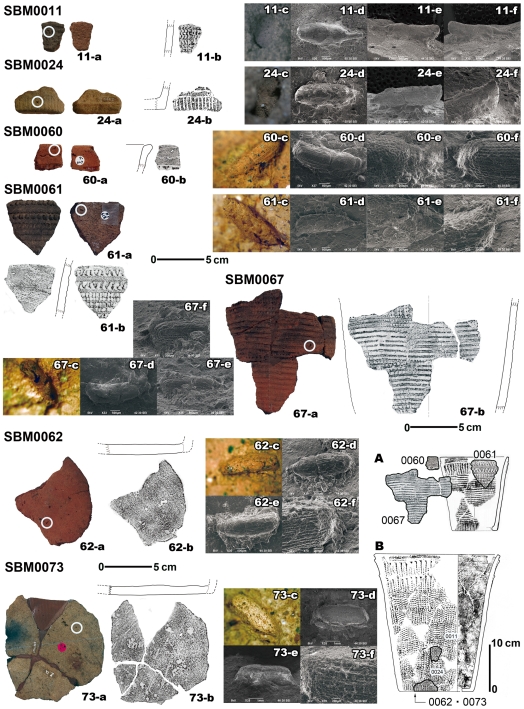
Scanning electron micrograph (SEM) images of the maize weevil impression replicas obtained from potsherds from the Sanbonmatsu Site. Maize weevil impressions on pottery (a), pottery section illustrations with rubbings (b), photos of cavities (c), and SEM images of the impression replicas (d,e,f). The white circles indicate the position of the cavities in the pottery. “A” and “B” show the positions of the potsherds with maize weevil impressions in pottery samples SBM0060, SBM0061, SBM0062, SBM0067, and SBM0073. “A “is from the Ohnakahara Site and “B” is from the Kakoinoharu Site in Kagoshima Prefecture. Details are provided in Table 2.

Adult granary weevils have elongated punctations on their thorax and other body parts, whereas adult rice and maize weevils have round or irregular punctations [20]. Most of the replicas lacked legs and a rostrum, but had round or irregularly shaped punctations, similar to those of maize weevils (Fig. 2, Table 2).

**Table 2:** Details of the maize weevil impressions from the Sanbonmatsu (SBM) site.

No.	Pottery type	Shape/part	Part of the weevil in the impression	Length of impression replicas (mm)	Estimated length of original weevil (mm)
SBM0011	Yoshida	Deep bowl/body	Ventral view (missing legs)	3.69	3.98
SBM0024	Yoshida	Deep bowl/rim	Ventral view (missing rostrum and legs)	3.32	4.58
SBM0060	Yoshida	Deep bowl/rim	Dorsal view (missing rostrum and legs)	3.11	4.33
SBM0061	Yoshida	Deep bowl/rim	Ventral view (missing legs)	4.11	5.41
SBM0062	Yoshida	Deep bowl/bottom	Dorsal view (missing rostrum)	3.45	4.69
SBM0067	Yoshida	Deep bowl/body	Side view	3.17	4.39
SBM0073	Yoshida	Deep bowl/bottom	Dorsal view (missing rostrum)	3.58	4.83

Samples are illustrated in Figure 2.

**Figure 3:**
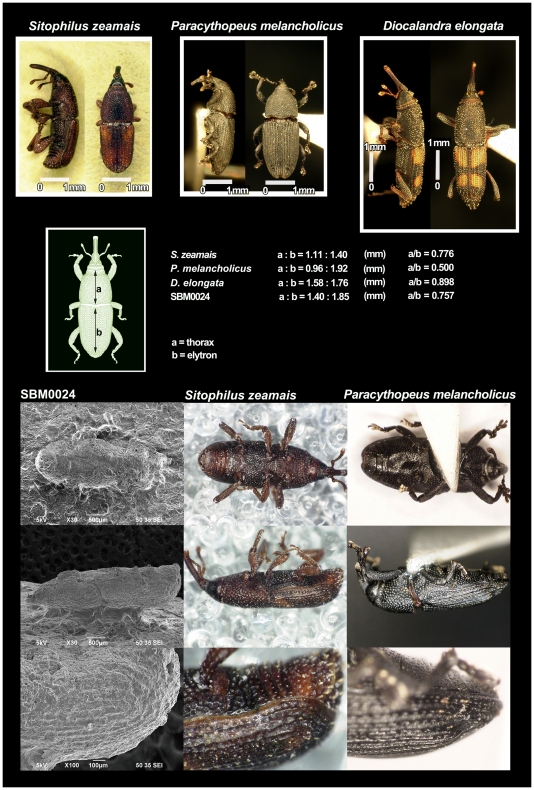
Diagnostic characteristics used to identify the weevil species. Three species of weevils (*Sitophilus zeamais, Diocalandra elongata*, and *Paracythopeus melancholicus*) are distinguishable by the ratio of thorax to elytron length. Another diagnostic criterion that distinguishes *S. zeamais* from *P*. *melancholicus* is the elytron end. Elytron does not cover the full abdomen in *S. zeamais* but extends the full length of the abdomen in *P*. *melancholicus* (bottom row of photographs).

However, two beetles are morphologically similar to maize weevils and should be excluded as possible explanations for the impressions: *Diocalandra* spp. and *Paracythopeus melancholicus* Roelofs. *Diocalandra* spp. are slenderer and longer than maize weevils [21]. The ratio of thorax to elytron length also differs among the three species: 0.898 for *Diocalandra* spp., 0.500 for *P*. *melancholicus*, and 0.757 for weevil impression replica SBM0024, which nearly equals the ratio of 0.776 for *S. zeamais* (Fig. 3). The side view of weevil impression SBM0024 is most similar to that of *S. zeamais* (Fig. 3). The diagnostic criterion that distinguishes *S. zeamais* from *P*. *melancholicus* is the elytron end, which is shorter than the abdomen in *S. zeamais* but covers the full length of abdomen in *P. melancholicus* (Fig. 3). These criteria can be seen clearly in the elytron end of the other weevil impression replicas (SBM0060, SMB0061, and SBM0062; Fig. 2), which suggest that the weevils from the Sanbonmatsu site were *S. zeamais*. These are therefore the oldest maize weevil relics in the world.

**Figure 4:**
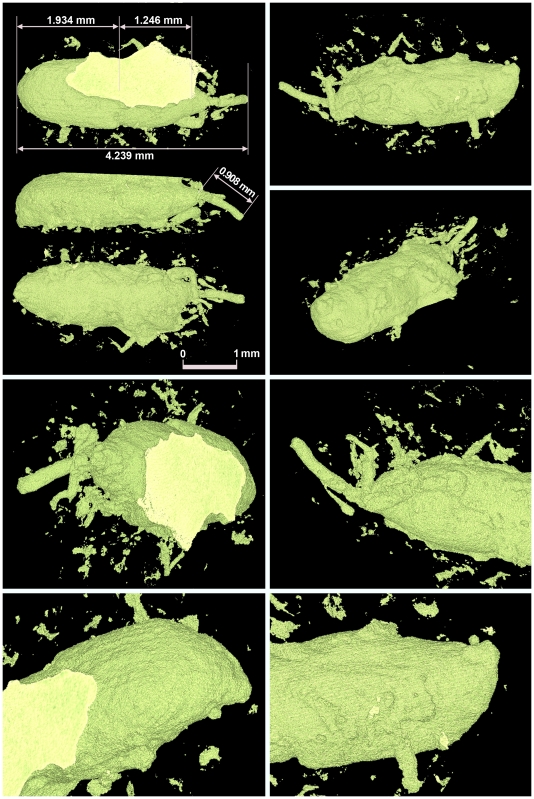
CT scan images of the impression of maize weevil SBM0024 from the Sanbonmatsu Site. The CT scan images show details of the insect's legs, rostrum end, and antennae that were previously unseen in the impression replicas. These findings demonstrate the method's superiority to the older impression replica method for correctly identifying insects. The lengths in this example are 0.908 mm (rostrum), 1.246 mm (thorax), and 1.934 mm (abdomen). In our experiment, the shrinkage of the mean lengths from the original maize weevils to the impressions they left in potsherd cavities were to 92.16% (rostrum), 91.63% (thorax), and 96.28% (abdomen) of the original length. Therefore, the original lengths would have been 0.985 mm (rostrum), 1.359 mm (thorax), and 2.008 mm (abdomen). These lengths are larger than those of modern reference specimens that were reared in cleaned rice grains (mean lengths of rostrum, thorax, and abdomen were 0.903, 1.105, and 1.428 mm, respectively; *n* = 20) and were similar to the size of weevils reared in chestnuts (mean lengths of rostrum, thorax, and abdomen were 1.056, 1.338, and 1.882 mm, respectively; *n* = 20).

To confirm this identification, we obtained CT scans of the impressions. These revealed details of the insect's legs, rostrum end, and antennae that have never previously been seen in weevil impression replicas (Fig. 4). These findings demonstrate the superiority of the CT scans compared with the original replica method for correctly identifying insects.

### Significance of maize weevils during the Earliest Jomon

Plarre [3] describes one accepted scenario for the evolution and propagation of grain pests, as follows. The currently existing *Sitophilus* species, including the three economically important grain pests discussed in this paper (*S. granarius*, *S. oryzae*, and *S. zeamais*), appear to be endemic to the forested areas of Asia. It therefore seems likely that the first infestation of stored grain occurred near the forested mountains of southwestern Asia. If these insects originally infested forest food sources such as acorns, the storage of these foods with cultivated grains would have provided the weevils with an alternative food source, and weevils capable of exploiting this resource would have had an advantage over other weevils, leading to co-evolution that produced weevils that were increasingly dependent on stored grain. Subsequently, this coevolution would promote the spread of these insects by humans.

Resarchers have not yet performed a phylogenetic analysis that would define the relationships among the various *Sitophilus* species. However, the high level of genetic similarity among the SOPE (*Sitophilus oryzae* primary endosymbiont) bacteria suggests that this endosymbiosis evolved only once, in a single original weevil species that subsequently evolved into *S. granarius*, *S. oryzae*, and *S. zeamais* [22]. Furthermore, dendrograms based on this DNA analysis suggest that these three *Sitophilus* species evolved from a common ancestor [23], [24].

Our new discovery supports this hypothesis and contradicts earlier hypotheses about the origin of these grain pests. We discovered seven new maize weevil impressions. This high discovery rate (one weevil impression replica per 2000 potsherds) is similar to or greater than that (one among 3000 to 20 000 potsherds) from the Late Jomon sites on Kyushu, indicating that these weevils were already house pests in the Earliest Jomon. These weevils are therefore the oldest house pests in the world, as they are older than the oldest archaeological records in Europe, which date to ca. 7000 BP [3]. Even if the propagation of *S. granarius* in Europe occurred during the diffusion of Neolithic agriculture, there is no evidence that demonstrates the existence of cereal agriculture in Japan or its arrival at that time. If the Late Jomon maize weevils were associated with the spread of rice or barley cultivation into Japan, then what is the significance of the Earliest Jomon records? Were other cereals cultivated in Japan at that time?

The Earliest Jomon is nearly synchronous with the beginning of rice cultivation in the Lower Yangtze River Valley of China, which is believed to be the origin of Asian rice cultivation [25]. However, there is currently no archaeological evidence that connects the two regions at that time. And the oldest evidence of barley and wheat cultivation in eastern Asia is younger than ca. 4000 BP. Thus, the archaeological evidence strongly suggests there were no cultivated cereals that could have been infested with maize weevils in Japan at ca. 10 500 BP. This suggests that the existence of maize weevils in the Earliest Jomon was not associated with rice cultivation. Nevertheless, the high weevil density at that time suggests the weevils were closely related to the Jomon people and lived in Jomon houses, where they fed on stored foods.

We do not know what kind of wild plant food they fed on or infested. Judging from the archaeobotanical data in Japan, we propose three candidates: the seeds of bamboo (Bambusoideae), acorns (*Quercus* spp.), and chestnuts (*Castanea* spp.). Bamboo seeds can be stored for sufficiently long periods to permit weevil development and have sometimes been used as an emergency food source during famines. Indeed, we found charred bamboo seeds at archaeological sites from the Late Jomon to the Ainu Culture period (from ca. 760 BP) on Hokkaido [26]. However, phytolith analysis suggests rapid decreases in bamboo flora in this region (Tanegashima Island) from 11 300 to 7300 BP because of the expansion of evergreen forest [27], [28]. On the other hand, acorns and chestnuts are important stored foods and were popular in the Jomon Period [12]. According to the short introduction by Delobel and Grenier [29], cereal weevils are polyphagous, and they successfully reared them in both acorns and chestnuts. Rice weevils prefer larger mature wheat kernels to smaller immature ones [30], and the body size (weight) of the three cereal weevils depends on the size of the plant seeds they infest [29]. Our preliminary experiment showed that adult weevils reared in acorns or chestnuts were about 1.24 times the size of those reared in cleaned rice. Figure 4 and Table 2 shows that the maize weevils at the Sanbonmatsu Site are roughly the same size as those that were reared in acorns and chestnuts. This suggests that bamboo seeds, acorns, or chestnuts might have been the stored foods that became infested by maize weevils during in the Jomon Period. However, additional research is required to provide more data and more accurate data to support that hypothesis.

### Do differences in the physiological characteristics and life cycle mean different origins?

Granary weevils and Japanese rice weevils cannot fly, unlike the maize weevil. Granary weevils and probably the Japanese rice weevil also depend strongly upon stored grain. Their complete larval development takes place inside the grains that they infest, and has not been observed in natural reservoirs [31], [20]. In other words, they are fully synanthropic grain pests and their spread depends upon the transport of infested grain or a suitable substrate [3]. In contrast, maize weevils shelter in winter under fallen leaves or in the soil [2]. After awakening in the spring, they feed on nectar from flowers. Their home range is within 400 m from human villages with grain storage facilities; thus, dispersal over longer distances requires human assistance (e.g., via grain transport) [26]. The pattern of spending most of their life outdoors is thought to result from an ancient adaptation to surviving on wild plants that produce suitable seeds [32]. Consequently, the maize weevil is clearly different from the granary weevil in terms of its degree of dependence on stored grain and in its life cycle.

Plarre [3] has suggested that the separation of *Sitophilus* lineages that led to the current grain pests must have occurred much earlier than previously believed. Therefore, our evidence supports Plarre's hypothesis; we believe that weevil pests in the Earliest Jomon in Japan must have evolved from a single common Asian progenitor. Initially, they would not have infested cereal grains (which were not stored at that time) but would instead have infested various kinds of wild plant seeds that were stored by the Jomon people. Our new discovery supports this hypothesis, and should encourage additional research on the ecology and history of maize weevils. In particular, more information is needed about when they began infesting stored food. This will require additional maize weevil specimens from other time periods and regions. No fossils of maize weevils or their ancestors have been discovered, so the origin and history of the taxon remain unclear despite our new evidence. In addition, explicit DNA analysis of modern grain weevils would provide important insights into their phylogenetic relationships.

## Materials and Methods

In our previous research, we obtained weevil impression replicas from several sites [11]. Then and in the present study, we began examinations of all potsherds obtained from the Sanbonmatsu Site in Kagoshima Prefecture in 2010. In February and April 2010, we found seven fragments that contained maize weevil impressions.

Weevil models were made from impressions in the clay of the recovered potsherds using the “impression replica” method introduced and adopted by Japanese archaeological researchers in the late 1970s. In this method, researchers inject silicone into a cavity (impression) in the clay, producing a model (replica) of the original form that created the cavity. However, the wet clay used to create a pot contracts as it dries and is fired in a kiln, and this compresses insect specimens. To determine the magnitude of the compression, which can then be used to reconstruct the original size of the trapped insects, we conducted an experiment in which we measured the rostrum, thorax, and abdomen of maize weevils (*n* = 20), then trapped them in wet clay similar in composition to the clay that would have been used to create the potsherds we recovered. Once the clay was dry and had been fired in a kiln, we measured the dimensions of the impression replicas, and calculated the shrinkage ratio as the impression replica size divided by the original size.

To allow a comparison of the impression replicas with modern grain weevils, we obtained data on modern weevils from my collection in Kumamoto City. The maize weevil resembles the rice weevil, but adult rice weevils are 2.1 to 2.9 mm long (mean, 2.3 mm), versus 2.3 to 3.5 mm (mean, 2.8 mm) for adult maize weevils [33].

To understand the developmental biology of the weevils, which would provide clues to their potential anthropogenic food sources, we reared maize weevils on many different foods. In total, we reared the insects on eight foods: rice, wheat, barley, adzuki bean, soybean, acorn, chestnut, and hemp. We found that the weevils could survive on rice, wheat, barley, acorn, and chestnut seeds. For weevils that survived to adulthood in rice, acorn, and chestnut, we measured their total length (*n* = 180) to determine whether the size of the food source affected their maturation and growth.

Although impression replicas from potsherds are important sources of information, they are difficult to examine because of their small size. To test whether modern technology could improve our ability to extract information from the impression replicas, we obtained scanning electron microscope (SEM) micrographs of the impression replicas and CT scans of the impressions. This work was performed at the Archaeological Center of Kumamoto University and the X-Earth Center of Kumamoto University, respectively, following each lab's standard protocols for scans of small objects.
